# Effects of robotic guidance on the coordination of locomotion

**DOI:** 10.1186/1743-0003-10-79

**Published:** 2013-07-19

**Authors:** Juan C Moreno, Filipe Barroso, Dario Farina, Leonardo Gizzi, Cristina Santos, Marco Molinari, José L Pons

**Affiliations:** 1 Bioengineering Group, Spanish National Research Council, CSIC, Carretera Campo Real, Madrid, Spain; 2 ASBG (Adaptive Systems Behaviour Group), Department of Industrial Electronics, University of Minho, Azurém, Guimarães, Portugal; 3 Department of Neurorehabilitation Engineering, Bernstein Focus Neurotechnology Göttingen, Bernstein Center for Computational Neuroscience, University Medical Center Göttingen, Georg-August University, Göttingen, Germany; 4 Pain Clinic -Center for Anesthesiology, Emergency and Intensive Care Medicine, University Medical Center Göttingen, Georg-August University, Göttingen, Germany; 5 Department of Movement and Sport Sciences, University of Rome “Foro Italico”, Rome, Italy; 6 Fondazione S. Lucia, Roma, Italy

## Abstract

**Background:**

Functional integration of motor activity patterns enables the production of coordinated movements, such as walking. The activation of muscles by weightened summation of activation signals has been demonstrated to represent the spatiotemporal components that determine motor behavior during walking. Exoskeleton robotic devices are now often used in the rehabilitation practice to assist physical therapy of individuals with neurological disorders. These devices are used to promote motor recovery by providing guidance force to the patients. The guidance should in principle lead to a muscle coordination similar to physiological human walking. However, the influence of robotic devices on locomotor patterns needs still to be characterized. The aim of this study was to analyze the effect of force guidance and gait speed on the modular organization of walking in a group of eight healthy subjects.

**Method:**

A group of healthy subjects walked on a treadmill with and without robotic aiding at speeds of 1.5, 2.0 and 2.5 Km/h. The guidance force was varied between 20%, 40%, 70% and 100% level of assistance. EMG recordings were obtained from seven leg muscles of the dominant leg and kinematic and kinetic features of the knee and hip joints were extracted.

**Results:**

Four motor modules were sufficient to represent the variety of behavioral goals demanded during robotic guidance, with similar relationships between muscle patterns and biomechanical parameters across subjects, confirming that the low-dimensional and impulsive control of human walking is maintained using robotic force guidance. The conditions of guidance force and speed that maintained correct and incorrect (not natural) modular control were identified.

**Conclusion:**

In neurologically intact subjects robotic-guided walking at various force guidance and speed levels does not alter the basic locomotor control and timing. This allows the design of robotic-aided rehabilitation strategies aimed at the modulation of motor modules, which are altered in stroke.

## Introduction

Control of locomotion has been largely studied in animal models, providing the background knowledge essential to the comprehension of the motor control in humans [1] and theories for training walking after neuronal damage. Spinal pattern generators, which are regulated by supraspinal control, have been regarded as the responsible for the locomotion both in humans and other vertebrates. Using fMRI, Jahn *et al.*[2] found evidence that the supraspinal network of quadrupeds is maintained in humans. Motor patterns are thought to be a result of interactions between the activity of the CNS and the peripheral inputs representing the biomechanical characteristics and the afferent sensorial activities [3].

Nowadays, the neurorehabilitation field has been adopting robotic devices to assist physical therapy on individuals with neurological disorders [4,5]. However, there is still a lack of basic knowledge on the effect of robotic gait training on human locomotion and its recovery in injured humans. Most theories supporting the conventional therapy techniques are based on data from experiments with animal models [6-9] and such theories have been transferred to design improved assisted gait training with robotic assistance. However, little is known on the neurobiological substrate of gait control in healthy humans to support the design of training strategies delivered with automated machines or robotic devices. This lack of knowledge is preventing the development of a sound and strong theoretical framework that is optimally suited to the robotic treatment of patients with injured brain or spinal cord [10,11].

Recent studies have been devoted to understand how the CNS orchestrates the neuronal responses corresponding to the planned movements, coordinating a large number of degrees of freedom of the musculoskeletal system [11-18]. The current evidence suggests that the nervous system controls complex motor tasks by using a low-dimensional combination of motor modules and activation signals [19,20]. This is particularly relevant for patient-cooperative strategies that allow patients to influence the timing of their leg movements [21].

In previous studies [12,16], the hypothesis that muscle activation patterns during walking are produced through the variable activation of a small set of motor modules (also called synergies) was tested by means of non-negative matrix factorization (NNMF) [22-25]. It has been proposed that human walking is mediated by muscle activations that can be expressed as the effect of few activation impulses at specific phases of the gait cycle delivered to muscle weightings [20].

The assist-as-needed control concept emerged to encourage the active motion of the patient. This approach is intended to manage simultaneous activation of efferent motor pathways and afferent sensory pathways during training. Zero-impedance control mode has been proposed to allow free movement of the segments. Also, the concept of a virtual tunnel that allows a range of free movement has been proposed [26]. However, such robotic devices need further research to show their suitability for walking training and their effects on over-ground gait [27-33].

Furthermore, it is not only important to assist as needed to correctly intervene but also to know what can be achieved by the available robotic tools. Results of a feasibility study supported the idea that a decentralized approach that explores the locomotor pattern of the patient can be effective in treatment of muscle spasticity after neurological damage [34]. The present study is directed to reveal the capacity of robotic force guidance and gait speed in affecting muscle synergies. According to our view, this information is essential for designing the correct reference and control systems to develop an assist-as-needed robotic rehabilitation protocol for walking. It can be argued that robotic-guided walking can be used to induce synergistic muscle activation patterns during walking that might be beneficial for the recovery of stroke survivors. Robotic guidance force (GF) is the amount of aid the patient receives. In a recent study, it has been concluded that walking in the Lokomat robotic trainer (Hocoma, Zurich, Switzerland) with minimal (0%) GF can be achieved by similar motor modules and activation signals as overground walking [22]. However, there is no evidence on the effect of adding a GF on the main modular organization of physiological walking in healthy humans. Therefore, the first hypothesis to be tested in this study is if using GF in robotic-aided walking alters the main impulsive synergistic structure of walking. The second hypothesis is that the GF and walking speed provided by the Lokomat gait trainer can be set in order to adequately shape the muscle weightings during human locomotion. Since these weightings are modified after a neurological lesion [20], verification of the second hypothesis would set the bases for designing rehabilitation strategies with robotic training. To verify the two hypotheses, healthy subjects walked at different speeds and GF percentages in the Lokomat gait orthosis.

## Methods

### Participants

Eight healthy participants (6 males and 2 females; age = 25.7 ± 4.4 years; body weight = 69.5 ± 9.8 kg; height = 1.76 ± 0.08 m) with no neurological injuries or gait disorders volunteered in the study. The participants had no previous experience with robotic-assisted walking. The local ethic committee (CSIC) provided ethical approval for this study.

### Procedures

By varying GF, the robot torque can be controlled from 0 to 100% and therefore, the amount of GF can be modulated to challenge the user. At 100% GF, the robot provides substantial assistance while at 0% GF, it does not assist the subject's leg movement and, therefore, it increases the demand of active participation.

At the beginning of the experiment, the robotic gait orthosis was adjusted to the patient’s anatomy. Hip width, length of upper and lower leg, size and position of the leg cuffs were individually adjusted to assure comfort. The range of motion was adapted to match a natural pattern preventing foot dragging, if needed. After being fitted and secured by a safety harness, the participants were asked to walk on the Lokomat robotic orthosis at speeds of 1.5, 2.0 and 2.5 km/h speed and robotic GF of 100%, 70%, 40% and 20% with a fixed body weight support (BWS) level of 30%. This value of BWS was chosen to enable comfortable walking with the robotic orthosis at high speeds. Moreover, it has been shown that changes in BWS do not alter significantly motor modules [16]. For assisting foot plantar flexion, foot lifters based on springs were present during the robotic aided walking. Each walking trial lasted 60 s. The participants were instructed to follow the robotic guidance aided by the Lokomat’s visual representation of biofeedback values. The visual biofeedback values, designed to motivate the patient to improve the walking performance [5], were displayed step-by-step in line graphs representing the walking performance over the last 10 steps.

The participants were instructed to follow the robotic movements in order to maintain a constant biofeedback value during each trial. All the combinations of speed and GF were recorded after a familiarization interval of 60 s for each combination. In addition, treadmill walking without the robotic orthosis and without BWS was measured for all participants at speeds of 1.5, 2.0 and 2.5 Km/h speed. The ten central gait cycles in each condition were selected for the analysis.

Bipolar electrodes (Ag-AgCl, Fiab S.p.A.) were mounted to record EMG signals from the rectus femoris (RF), vastus lateralis (VL), semitendinosus (ST), biceps femoris (BF), gastrocnemius medialis (GM), gastrocnemius lateralis (GL), and tibialis anterior (TA) of the dominant leg of each participant, using a wireless EMG acquisition system (BTS Pocket EMG, Myolab) with a sampling rate of 1 KHz. Electrode sites were determined following the SENIAM [35] recommendations. The skin was shaved and cleaned with alcohol prior to electrode positioning. The data were wirelessly streamed during the treadmill and robotic-guided walking conditions and analyzed using Matlab 7.0 (The Mathworks, Natick, MA) and SPSS statistical software (v. 18.0 IBM).

In the robotic-guided walking condition, the knee and hip angles in the sagittal plane and the forces exchanged against the machine at the knee and hip joints were recorded from the analog output of the Lokomat. In the treadmill walking condition (walking without the aid of Lokomat), an electrogoniometer was used to measure the knee joint angle in the sagittal plane. In all conditions, a foot switch was placed beneath the heel of the dominant leg to identify and segment the gait cycles. The values of the visual biofeedback from the Lokomat were recorded for every gait cycle and used for offline validation of each trial.

### EMG signal analysis

The raw EMG data were band-pass filtered (3^rd^ order Butterworth digital, bandwidth 20–400 Hz, roll-off rate of 12 dB/decade) to attenuate DC offset, motion artifacts, and high frequency noise. The EMG signals were rectified and were smoothed using a 50-point root mean squared (RMS) algorithm. The smoothed EMG signals were interpolated per each stride cycle in order to obtain average stride cycles with 101 points. Stride cycles were then averaged to obtain time-normalized gait cycles with 101 points. For each muscle and participant, each time-normalized EMG signal was amplitude-normalized by its maximal value obtained in all the conditions of speed and GF. Although averaging of EMG waveforms decreases the variability of the signal, inter-trial variability is reduced in the stereotyped muscular activity in the Lokomat [22]. These normalized EMG signals were computed to obtain the average of the group, for each muscle and condition of speed and GF, in order to assess the structure of control rather than the precise weights of individual muscles. For each subject and for the average of the group, the EMG signals of each condition were combined into an *m* x *t* matrix (EMG_0_), where *m* indicates the number of muscles (seven muscles in this case) and *t* is the time base (101 values that represents the gait cycle from 0% until 100%) [9].

An NNMF algorithm [22] was applied to the EMG_0_ matrix for the extraction of motor modules from each subject in each condition. The number of modules and activation signals, *n*, was varied between two, three, four and five, and the NNMF algorithm found the properties of the modules by updating two matrices: an *m* × *n* matrix, which specifies the relative weighting (motor modules) of a muscle in each activation signal, and an *n* × *t* matrix, which specifies the activation timing of each activation signal. These two matrices were multiplied to produce an *m* × *t* matrix (EMG_r_) in an attempt to reconstruct the EMG signals. EMG_r_ was compared to EMG_0_ by calculating

(1) $$\overset{m}{\underset{i=1}{\sum }}\overset{t}{\underset{j=1}{\sum }}{\left(\mathrm{EM}G_{0}\left(i,j\right)-\mathrm{EM}G_{r}\left(i,j\right)\right)}^{2}$$

The result was used for iterative optimization until a local minima was found on the motor modules and the activation signals that minimized the error.

The variability accounted for (VAF) was calculated to determine the minimum number of activation signals needed to adequately reconstruct EMG_0_ of each subject and of the average of the group. The VAF was calculated as the ratio of the sum of the squared error values to the sum of the squared EMG_0_ values, as follows:

(2) $$\mathrm{VAF}=1-\frac{\sum_{i=1}^{m}\sum_{j=1}^{t}{\left(\mathrm{EM}G_{0}\left(i,j\right)-\mathrm{EM}G_{r}\left(i,j\right)\right)}^{2}}{\sum_{i=1}^{m}\sum_{j=1}^{t}\frac{m}{i=1}\frac{t}{j=1}{\left(\mathrm{EM}G_{0}\left(i,j\right)\right)}^{2}}$$

VAF was calculated for each muscle and for each condition within the gait cycle. In order to ensure the quality of reconstructed signals within each region of the gait cycle, VAF was also calculated within seven phases [9] of the gait cycle: 1) initial double support, 2) mid stance, 3) terminal stance, 4) pre swing, 5) initial swing, 6) mid swing, and 7) terminal swing. We analyzed the VAF results from the computed activation signals from the average EMG of the group.

In order to visually analyze the possible existence of shared motor modules for all conditions, the activation signals were also computed reconstructing the signal by means of the same motor modules [36] (those obtained in treadmill walking using 2.5 Km/h speed) for all conditions.

The percentage contribution of 7 different periods (gait subphases) to total muscle activity (EMG envelopes) and activation signals during stance and swing was calculated for all combinations of GF and speed. This separation was used to compute the contribution of muscular activation signals for statistical comparison of activation signals between treadmill and robotic walking. Thus, activation signals were investigated by calculating the integral of the signal amplitude for the period of each subphase of the gait cycle. These integrals over the 7 intervals are related to the timing of muscle activation which was compared between normal and treadmill walking.

### Kinematic and force analysis

The kinematic and force data were averaged per each stride in order to obtain data time normalized, expressed as a percentage of the total gait cycle, i.e., 0 to 100%.

The angular range of motion (ROM) in the sagittal plane for hip and knee was computed by subtracting the minimum joint angle from the maximum joint angle for Lokomat trials for each condition of GF and speed. The ROM in the sagittal plane for knee during the treadmill walking was also calculated, for each speed. The time (% of gait cycle) at which the minimum and maximum angles occurred were also determined.

The kinetic range of forces (ROF) in the hip and knee joints of the Lokomat was found by subtracting the minimum joint force from the maximum joint force for robotic-guided walking trials for each condition of GF and speed and also for each gait phase.

### Statistical analysis

The differences in motor modules and activation signals across subjects for treadmill and robotic-guided walking, and among subjects in robotic-assisted walking were tested using a three-way ANOVA and Tukey’s post hoc analysis. The activation signals were computed reconstructing the signal by means of the same motor modules (those obtained in treadmill walking using 2.5 km/h speed) for all conditions in order to test uniform modular organization for all conditions of walking.

The consistency of the activation signals between robotic and treadmill walking - at the same speeds- was tested with a Pearsons’s correlation analysis of the integrals in the 7 intervals during the gait cycle.

## Results

### Muscle activations

**Figure 1:**
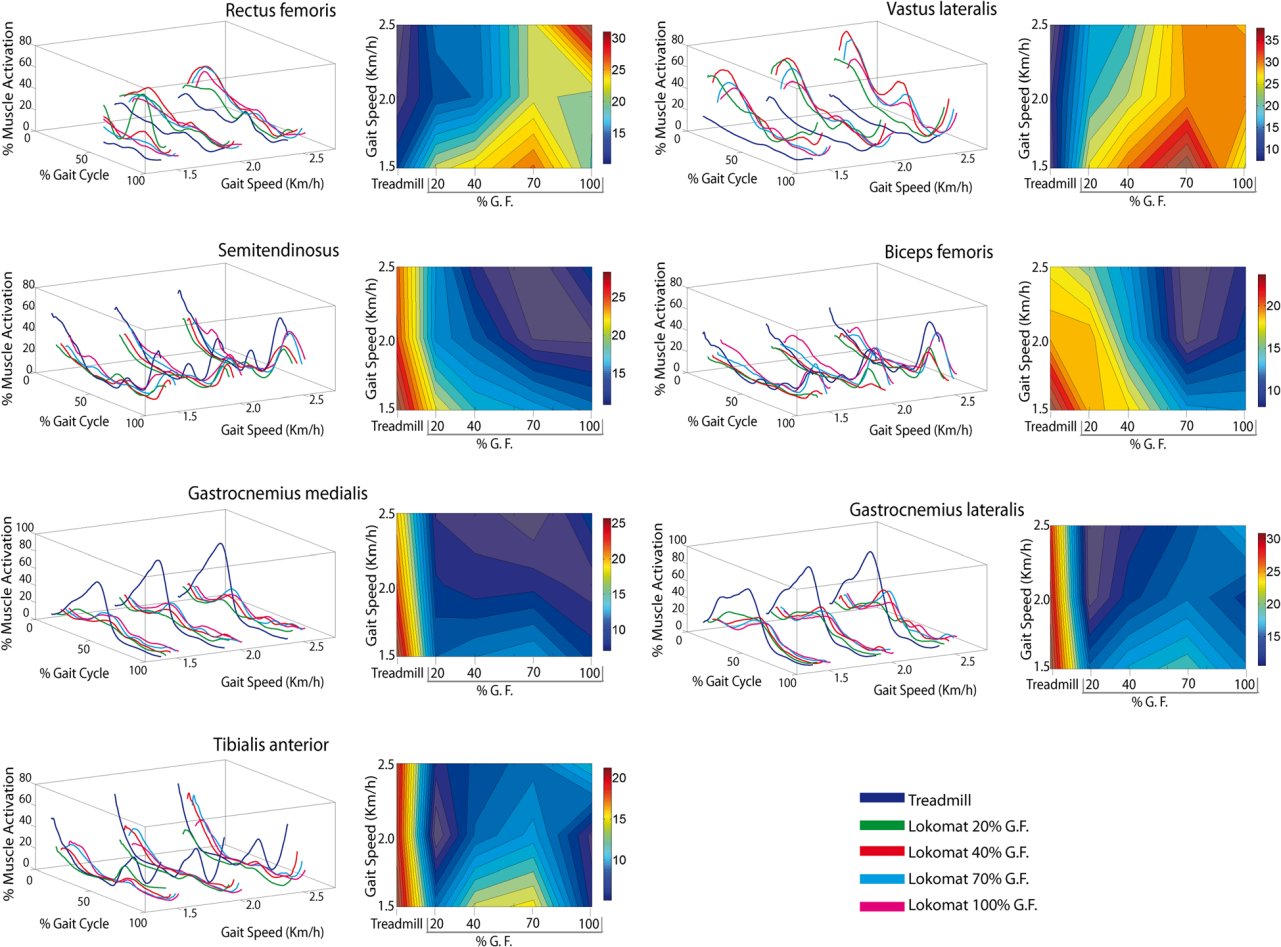
**Dependence of muscular activation on the level of robotic GF and walking speed for each investigated muscle.** Left panel: group average activation is represented for treadmill and robotic walking (free walking on a treadmill and walking with four levels of GF in the robot-aided condition) against speed. Right panel: the integral of the average EMG envelopes are represented in a contour plot with 20 levels; interpolation was done to represent walking speed with respect to the treadmill (unassisted) and robotic walking (four levels of GF) conditions.

The average EMG recorded from each muscle across subjects for all conditions is illustrated in Figure 1. Significant variations were found according to the demand. In general, mean muscle activations were found to be increasing with an increase in walking speed, for all percentage of GF (Figure 1). In particular, it was observed that across walking speeds the muscle activation was significantly increased for 20% and 40% GF if compared to other GFs.

The quadriceps muscles during robotic-guided walking contributed with greater activity than during treadmill walking, for all GF levels. It can be observed that GM and TA muscles contributed less significantly to the mechanical demand imposed during robotic-guided walking. The activation of the hamstrings muscles was in general similar for all the conditions although a generalized reduction of activity was observed during the transition to the swing phase during robotic-guided walking.

Partial contributions at gait phase of recorded muscles to the total muscle activity per stride, revealed the highest correlation for VL, ST and BF, when comparing treadmill to robotic-guided walking.

### Robotic-guided walking kinematics and forces

**Figure 2:**
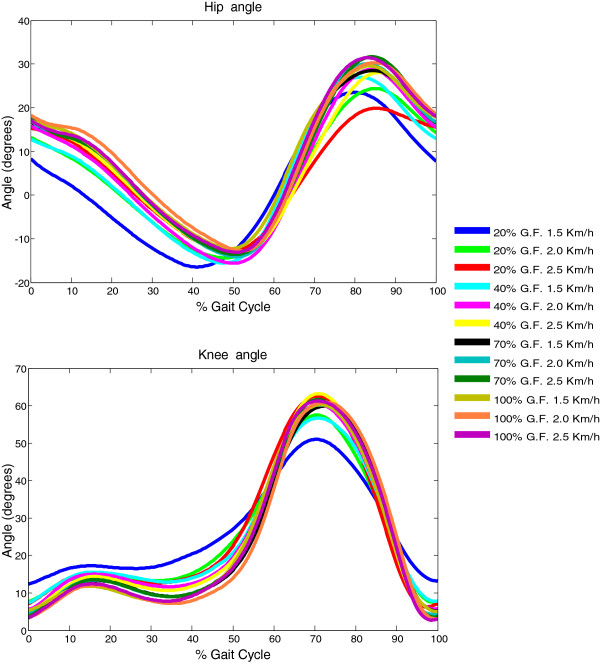
Average kinematic trajectories of the hip and knee joints (sagittal) during the gait cycle in the robotic-guided walking condition.

To determine whether the subjects modified the joint trajectories in response to the altered mechanical demand, we examined the average knee and hip joints trajectories and ROM. The resulting angular patterns and ROM (sagittal) of the hip and knee joints during the robotic-guided condition were examined. Figure 2 illustrates the average knee and hip angular trajectories, pooled for each testing conditions. The angular pattern and ROM of the knee shows a common pattern of trajectory during all conditions, as no significant differences were found. Although the robotic exoskeleton guides the joints of the limb subjects through pre-programmed trajectories, a small amount of variance was found on the pooled trajectories, which in general increased with decreasing the amount of GF.

The ROM at the hip with 20% GF, reduced with increasing GF, regardless of speed. The ROM at the knee with 20% GF and 1.5 Km/h speed was significantly reduced when compared to other combinations. It should be considered that variations in ROM that resulted from variations in walking speed may be explained by the dependence of the trajectory on walking speed in the Lokomat robot. In general, both the knee and hip joints ROM increased with increasing speed and GF, except for the condition with 20% GF, in which the hip ROM decreased with increasing speed.

**Figure 3:**
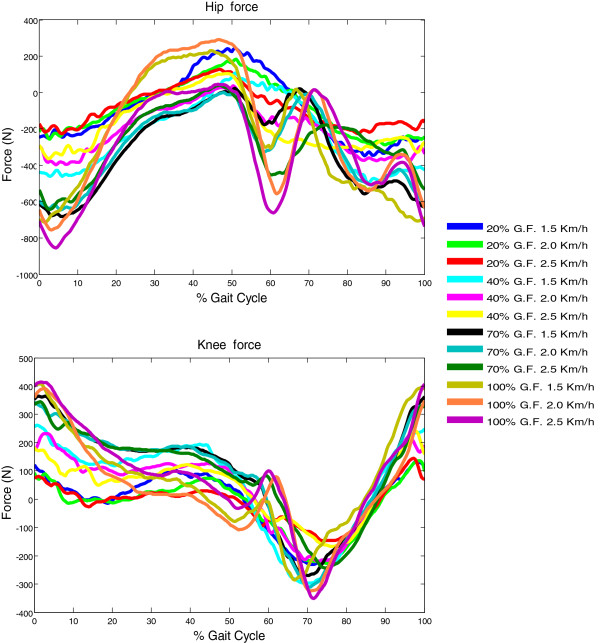
Average joint forces during the gait cycle in the robotic-guided walking condition.

To determine whether the subjects modified the patterns of joint forces during the gait cycle, we examined the average knee and hip exoskeleton joint forces. In general, the subjects were able to walk with a similar kinematic pattern imposed by the robot but changes in the mechanical pattern were observed (Figure 3). The ROF were decreased with the decrease of GF and the increase of speed.

The main deviations across combinations in the interaction forces were found in the transition from stance to swing phases. For the hip joint, we observed that with 20% and 40% GF, as the leg moved to prepare the swing motion and initiate it, the relative hip extension and flexion forces were small. Nevertheless, for higher GF (70% and 100%), the hip force patterns required a more complex strategy as subjects exerted significantly higher hip flexion forces at mid-swing. This reveals a strategy that is adopted to pull the leg towards swing that is accentuated with augmented mechanical demand [37] (given the instruction to follow the robotic guidance aided by visual representation of biofeedback values). This behavior correlates with the increased RF (hip flexor) activity and decreased activity of the hamstrings (hip extensor).

For the knee joint, the ROF decreased with the decrease of GF and the increase of speed. The ROF using 20% and 40% GF was reduced when compared to higher levels of GF. The main differences in forces across combinations for this joint were observed in the transition from stance to swing. For 20% and 40% GF, the limb produced reduced extension torques during pre-swing, followed by reduced flexion torques at initial swing. In turn, using 70% and 100% GF resulted in increased knee extension torques at pre-swing followed by increased knee flexion torques at initial swing.

### Motor modules

**Figure 4:**
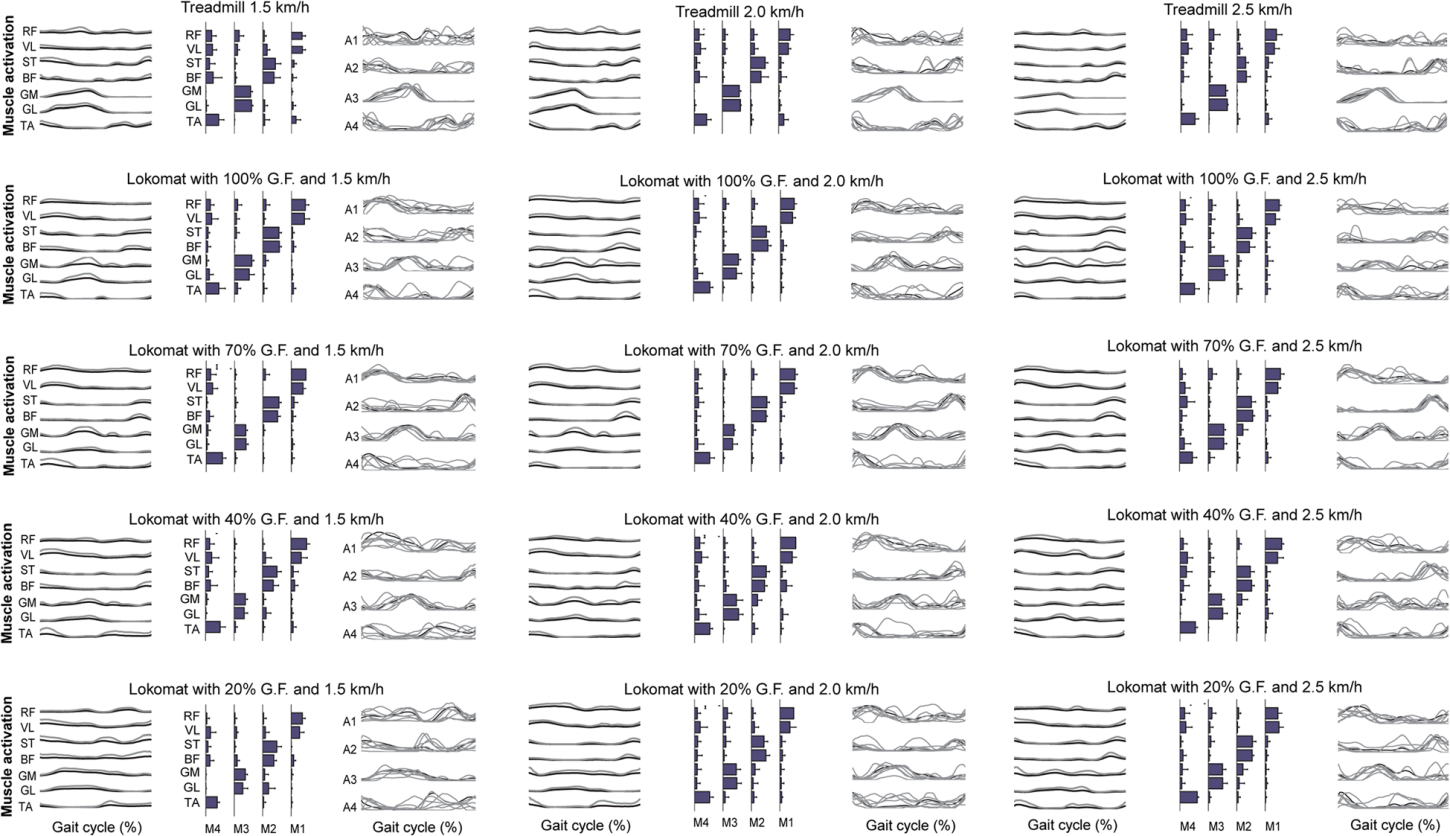
**Four modules are sufficient to reconstruct the EMG envelopes of all the testing conditions with a VAF higher than 90%.** Representation of this modular control is organized in three columns, one for each gait speed. Average and standard deviation of the EMG envelopes of the seven muscles (left). Average and standard deviation of motor modules (center). Activation signals (right), with thin gray lines representing the results of each subject of the study, and thick black lines representing the group average.

A minimal VAF value of 80% in each gait cycle portion was required to consider the reconstruction quality satisfactory. Preliminary tests led to exclude dimensionality five since inclusion of a 5^th^ module did not improve substantially the reconstruction quality. Four motor modules accounted for robot-aided walking with VAF above 80% for all muscles and gait phases. The computed motor modules, activation signals and EMG envelopes for all conditions of GF and speed are represented in Figure 4. Module 1 consisted mainly of flexor activity from the RF (hip flexor, also knee extensor) and activity of the VL (mainly a knee extensor). This module was mainly active during the midstance phase. Module 2 mostly consisted of activity of the ST (knee flexor) and BF (hip extensor) muscles at terminal swing and midstance. Module 3 consisted mainly of activity of the GM and GL (ankle plantarflexors) and this module was primarily active during late stance. Module 4 consisted mainly of activity of the TA (ankle dorsiflexor). This module was mainly active during midstance and along the swing phase.

### Modular control in treadmill walking

The calculated motor modules on treadmill walking confirmed the assumption that low-dimensional organization is present and similar among subjects and speeds (no significant difference, Additional file 1: Table S1). The test for dependent variables confirmed no significant differences in activation signals among subjects (P > 0.05) and no significant difference between speeds (P > 0.05).

### Modular control in robotic-guided walking

The calculated motor modules during robot-aided walking were similar among subjects (Additional file 1: Table S1). Results showed that activation signals are quite different among subjects, for the same conditions of GF and speed (P = 0.03). Activation signals shown to be significantly different for variations of speed across GF conditions (P < 0.05). The results showed that motor modules on the robot-aided walking condition were similar for each subject between conditions (P > 0.05). The calculated average of motor modules among subjects reflected high similarity for all conditions.

From the correlation analysis of activation signals, the robotic-guided walking using 20% GF and 1.5 Km/h speed resulted in the lowest similarity with respect to the other conditions (Additional file 1: Tables S1-S4). This result was confirmed by the fact that the subjects reported discomfort during this condition. Robot-aided walking with 100% GF resulted in the lower similarity with the treadmill walking condition. As for all the conditions of robot-aided walking with GFs of 40% and 20% we found significantly high similarities with respect to treadmill walking, except for the combination of 20% GF and 1.5 Km/h speed.

**Figure 5:**
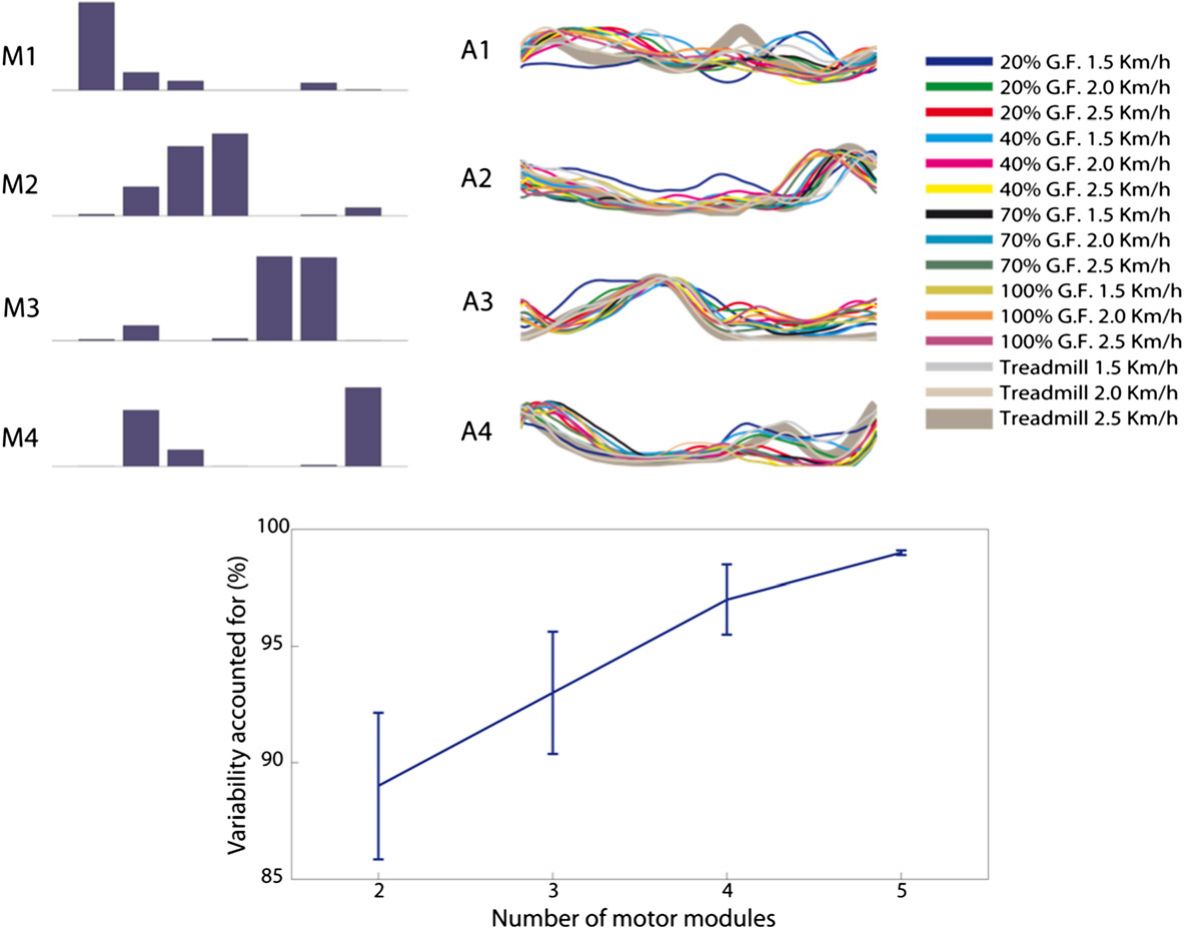
**On/off timing patterns of the four activation signals within a gait cycle for the different conditions of speed and mechanical constraints.** Threshold definition for activation signal onset was to set the activation signal ON when the activity exceeds the triple SD range. Average knee angle profile within a gait cycle is presented for reference (top).

The robot-aided condition of 20% GF and 1.5 Km/h speed was characterized by significantly different timing of activations (Figure 4 and Figure 5). The motor modules exhibited remarkable changes during 20% GF at 1.5 Km/h speed condition with respect to all experimental conditions (Tables 1, 2 and 3) for all subjects.

**Table 1:** Kinematic trajectories of the hip and knee joints in the sagittal plane during robotic-guided walking

		**Hip**						**Knee**					
		**Min (1)**	**Max (2)**	**ROM (3)**	**SD (4)**	**Time (Min) (5)**	**Time (Max) (6)**	**Min (1)**	**Max (2)**	**ROM (3)**	**SD (4)**	**Time (Min) (5)**	**Time (Max) (6)**
20% GF	1.5 Km/h speed	−16,47	23,49	39,96	14,10	41,20	80,40	12,40	51,02	38,62	11,76	0,40	70,40
	2.0 Km/h speed	−14,37	24,38	38,75	11,64	48,40	85,20	7,56	57,52	49,96	13,29	99,20	70,80
	2.5 Km/h speed	−13,01	19,86	32,87	8,42	49,20	85,60	6,25	62,52	56,27	15,07	97,60	70,80
40% GF	1.5 Km/h speed	−15,55	26,99	42,53	10,91	48,00	81,60	7,33	56,68	49,35	11,21	0,40	71,20
	2.0 Km/h speed	−15,60	28,77	44,36	7,91	50,40	84,40	5,67	60,39	54,72	12,27	0,40	71,20
	2.5 Km/h speed	−13,79	28,07	41,85	5,78	51,20	86,40	3,99	63,24	59,26	11,23	98,80	71,20
70% GF	1.5 Km/h speed	−13,90	28,48	42,38	6,27	51,60	84,80	5,02	59,97	54,95	9,82	0,40	73,20
	2.0 Km/h speed	−14,12	30,00	44,12	5,06	51,20	83,20	4,31	60,18	55,86	9,12	98,80	71,60
	2.5 Km/h speed	−13,60	31,67	45,28	6,54	50,80	84,80	3,52	61,65	58,13	10,23	99,20	71,20
100% GF	1.5 Km/h speed	−12,29	29,54	41,83	7,13	49,60	84,00	4,97	60,32	55,35	11,42	100,40	72,00
	2.0 Km/h speed	−12,80	30,28	43,08	3,92	52,80	85,60	3,11	60,92	57,81	7,04	100,00	72,80
	2.5 Km/h speed	−13,08	31,44	44,52	2,42	51,20	83,60	2,75	61,26	58,51	5,99	98,80	71,20
(1)	Minimum angle												
(2)	Maximum angle												
(3)	Range of motion ( (2) - (1) )												
(4)	Standard deviation												
(5)	Correspondent % Gait cycle of the minimum												
(6)	Correspondent % Gait cycle of the maximum												

Minimum and maximum angles (with corresponding timing), ROM and standard deviations for the average group.

**Table 2:** Joint forces in the sagittal plane during robotic-guided walking

		**A) Hip kinetics - Forces actuating in robotic hip joint**														
		**Phase 1 initial double support (0-10% GC)**		**Phase 2 mid stance (10-30% GC)**		**Phase 3 terminal stance (30-50% GC)**		**Phase 4 preswing (50-60% GC)**		**Phase 5 initial swing (60-73% GC)**		**Phase 6 mid swing (73-87% GC)**		**Phase 7 terminal swing (87-100% GC)**		**ROF (1)**
		**Min.**	**Max.**	**Min.**	**Max.**	**Min.**	**Max.**	**Min.**	**Max.**	**Min.**	**Max.**	**Min.**	**Max.**	**Min.**	**Max.**	
20% GF	1.5 Km/h speed	−252	−206	−229	−12	−9	240	117	244	−194	110	−349	−202	−340	−245	593
	2.0 Km/h speed	−257	−193	−195	−18	−11	172	41	182	−126	45	−287	−130	−288	−238	470
	2.5 Km/h speed	−228	−173	−200	−1	3	129	−61	96	−171	−43	−226	−161	−204	−153	357
40% GF	1.5 Km/h speed	−460	−428	−444	−160	−155	85	13	107	−184	37	−497	−195	−483	−410	604
	2.0 Km/h speed	−397	−298	−384	−95	−93	31	−171	36	−191	−134	−374	−200	−377	−296	433
	2.5 Km/h speed	−364	−290	−324	−22	−20	104	−214	89	−292	−221	−315	−287	−328	−246	468
70% GF	1.5 Km/h speed	−683	−610	−639	−158	−151	6	−177	24	−168	21	−557	−165	−632	−485	707
	2.0 Km/h speed	−645	−592	−601	−133	−130	0	−325	−6	−304	−9	−508	−98	−615	−423	645
	2.5 Km/h speed	−643	−535	−551	−94	−90	39	−453	−11	−454	−190	−287	−178	−533	−295	682
100% GF	1.5 Km/h speed	−714	−518	−507	152	160	232	−306	157	−316	18	−535	−336	−707	−536	946
	2.0 Km/h speed	−756	−591	−572	185	195	291	−479	261	−559	14	−541	−17	−636	−339	1046
	2.5 Km/h speed	−854	−694	−680	−25	−21	45	−654	7	−662	15	−507	−18	−738	−385	899
		**B) Knee Kinetics - Forces actuating in robotic knee joint**														
		**Phase 1 initial double support (0-10% GC)**		**Phase 2 mid stance (10-30% GC)**		**Phase 3 terminal stance (30-50% GC)**		**Phase 4 preswing (50-60% GC)**		**Phase 5 initial swing (60-73% GC)**		**Phase 6 mid swing (73-87% GC)**		**Phase 7 terminal swing (87-100% GC)**		**ROF (1)**
		**Min.**	**Max.**	**Min.**	**Max.**	**Min.**	**Max.**	**Min.**	**Max.**	**Min.**	**Max.**	**Min.**	**Max.**	**Min.**	**Max.**	
20% GF	1.5 Km/h speed	28,4	121	−14	70,3	47,1	103	−110	45,4	−232	−116	−225	−33	−27	135	367
	2.0 Km/h speed	−14	93,2	−13	27	24,1	74,6	−77	31,1	−221	−79	−227	−47	−42	136	363
	2.5 Km/h speed	20,3	80,3	−27	23,1	7,53	30,2	−95	8,08	−138	−62	−148	−35	−30	144	292
40% GF	1.5 Km/h speed	156	262	118	152	102	195	−138	94,2	−297	−150	−283	8,94	24,9	249	559
	2.0 Km/h speed	143	232	86,5	139	97,1	127	−105	89	−219	−101	−218	−12	0,22	220	451
	2.5 Km/h speed	83,5	185	49,4	117	77,6	121	−66	75,4	−152	−61	−168	−18	−8,8	242	410
70% GF	1.5 Km/h speed	251	364	169	246	124	183	−25	117	−270	−37	−240	−8,5	1,09	362	633
	2.0 Km/h speed	250	343	173	243	116	187	−23	109	−311	−42	−286	6,97	13,2	333	654
	2.5 Km/h speed	248	344	172	249	87,7	172	49,9	84,5	−226	68,3	−244	−36	−26	330	588
100% GF	1.5 Km/h speed	236	408	74,6	229	−68	76,9	−79	−2,6	−286	−23	−165	50,7	62,5	394	695
	2.0 Km/h speed	256	392	19,9	243	−88	17,9	−108	37,4	−324	79,7	−308	−12	−5,4	348	716
	2.5 Km/h speed	286	415	88,2	281	−5,4	98,5	−33	101	−351	98	−324	33,9	43,2	407	766
(1) ROF is the range of forces																

Minimum and maximum forces and ROF per gait phase for each condition.

**Table 3:** Values of correlations of contributions of activation signals along the gait cycle between treadmill and robotic walking computed for the same velocity of walking

		**Lokomat 20% G.F.**	**Lokomat 40% G.F.**	**Lokomat 70% G.F.**	**Lokomat 100% G.F.**
Activation signal 1	Treadmill 1.5 Km/h	0.55	0.76*	0.75	0.72
	Treadmill 2.0 Km/h	0.88**	0.83*	0.86*	0.83^*^
	Treadmill 2.5 Km/h	0.68	0.84*	0.84*	0.80^*^
Activation signal 2	Treadmill 1.5 Km/h	0.90**	0.90**	0.41	0.57
	Treadmill 2.0 Km/h	0.88**	0.88**	0.75^*^	0.68
	Treadmill 2.5 Km/h	0.84*	0.55	0.63	0.63
Activation signal 3	Treadmill 1.5 Km/h	0.93**	0.99^**^	0.96**	0.98**
	Treadmill 2.0 Km/h	0.97**	0.99^**^	0.98**	0.93**
	Treadmill 2.5 Km/h	0.90**	0.97^**^	0.98**	0.98**
Activation signal 4	Treadmill 1.5 Km/h	0.41	0.78^*^	0.06	0.06
	Treadmill 2.0 Km/h	0.91**	0.13	0.26	0.08
	Treadmill 2.5 Km/h	0.73	0.72	0.75*	0.75

*Correlation is significant at the 0.05 level (2-tailed).

**Correlation is significant at the 0.01 level (2-tailed).

**Figure 6:**
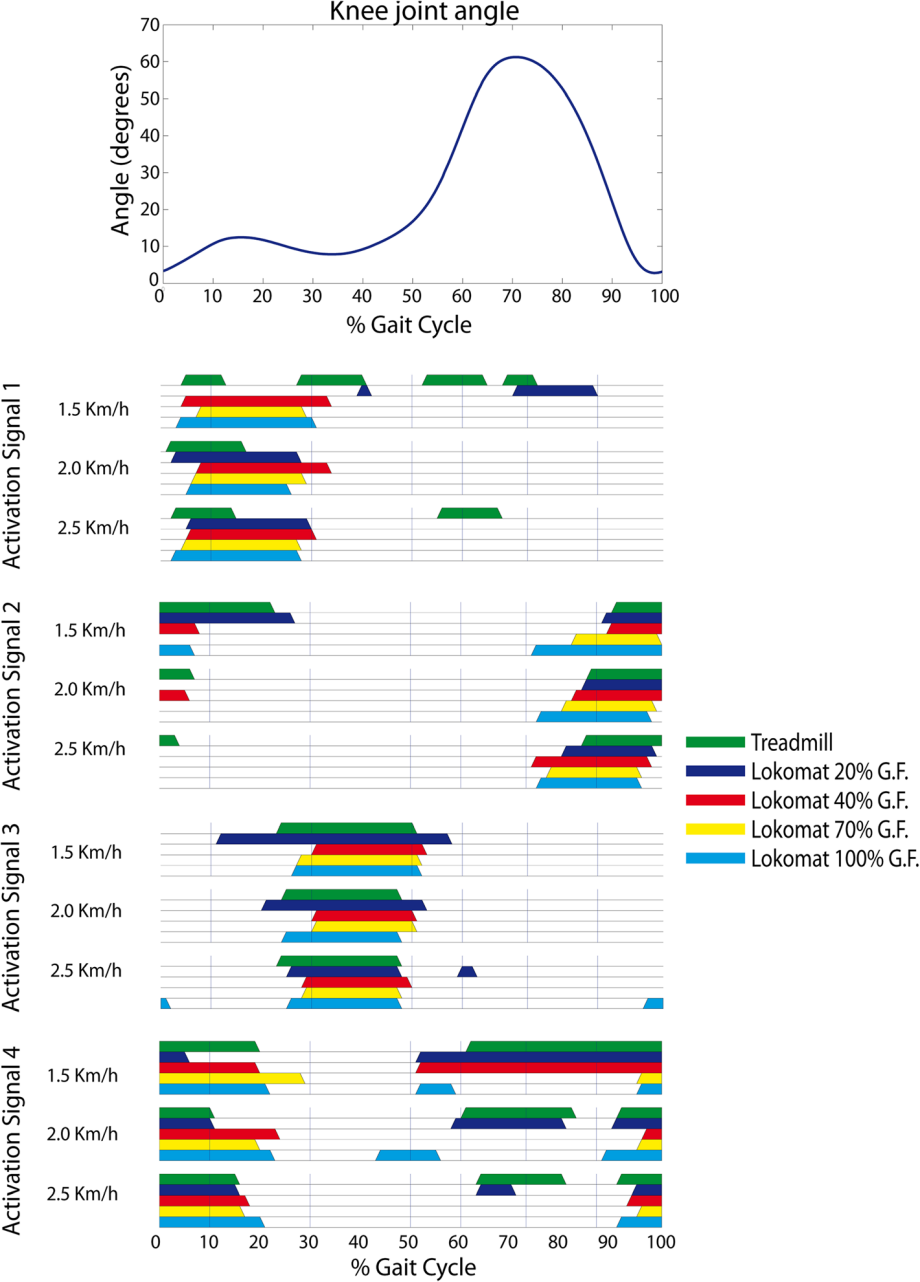
**TOP: motor modules (M) and average computed activation signals (A) of all conditions of GF and speed (right) tested with a fixed matrix of modules (left) (2.5 Km/h speed).** BOTTOM: Group average of variability accounted for (VAF) according to the number of motor modules. Means ± SD of VAF for the seven gait phases and for all the conditions investigated. Four modules are sufficient to reconstruct the EMG envelopes of all the testing conditions with a VAF higher than 90% for all muscles and gait sub-phases.

We tested the computation of activation signals with fixed modules (Figure 6) across conditions. From this analysis, the activation signal 1 was similar across all 15 combinations, the activation signal 2 was similar for treadmill walking and robotic-guided walking using 20% and 40% GF at low speeds, the activation signal 3 was similar between treadmill and robot-aided walking for all conditions with GF > 20%, and the activation signal 4 was similar between treadmill and robot-aided walking at 20% GF and low speeds.

We tested the consistency of the reconstructed activation signals between robotic and treadmill walking (Table 3) by determining if strength of contribution (association estimated with correlation analysis) of activation signals is maintained with changes in GF for the same speeds. From this analysis, it is observed that timing of activation signals shows in general small differences between the two conditions, as can be observed in the overview of the activation patterns with on/off timing patterns along the gait cycle (Figure 5). In particular, the timing of activation signals is highly or at least moderately maintained in robot-aided walking at 2 and 2.5 km/h (Table 3), except for the activation signal 4 at 40% GF, 70% GF and 100% GF.

We computed the total variability accounted for all muscles, conditions and gait sub-phases based on the number of motor modules extracted. The variability accounted for by 4 motor modules was >90% for the average of all muscles, conditions and gait sub-phases (Figure 5). A lower number of modules would not ensure that the modular representation is able to cope with the complete set of kinematic and dynamic constraints introduced by the robot during our testing conditions.

## Discussion

We investigated the effect of GF when walking with an exoskeleton on the muscular activation patterns and biomechanical parameters of healthy humans. The findings indicate that a low-dimensional and impulsive control of human walking is maintained with variations of robotic GF, despite changes in muscle weightings. It has been concluded that in neurologically intact subjects robotic-guided walking at various GF levels does not alter the basic locomotor control and timing of muscular activation patterns.

Recent studies have provided evidence of a modular control of synergistic lower limb muscle groups during locomotion of healthy [36-39] and subjects with neurological damage [22]. A simulation-based study reported changes in the modular control with specific biomechanical tasks using emulated subject’s responses [38].

Understanding how the CNS coordinates the muscle activity during robotic-guided walking is crucial for the design of the robotic therapy [40]. In a recent study, it was concluded that motor modules observed in subacute stroke patients during locomotion are different from those used by healthy controls, despite similar impulsive activation signals [22]. Also, alterations of the muscle activation patterns during robotic-guided with respect to treadmill walking in healthy subjects with fixed mechanical demand, have been reported [27].

The experimental protocol in the present study was designed to test and characterize the effects on the modular control of walking, muscle activations and biomechanics of the variations on mechanical demand imposed by a motorized exoskeleton. Our focus was on guidance force and velocity whilst the effects of body weight support have been reported elsewhere [29]. We are currently investigating the effects of biofeedback on the neuromuscular patterns during robotic walking with stroke survivors.

Four motor modules were sufficient to describe the muscular activations for all recorded muscles in all subjects and across conditions. It has been concluded that similar motor modules and activation signals are extracted from robotic walking at 30% BWS and overground walking for the included pool of healthy subjects [22]. The experimental data also revealed similar relationships between motor modules and biomechanical parameters across subjects. This gives the support to analyze and characterize the effects of robotic guidance on the coordination of lower limb muscles during locomotion. The main characteristic roles of motor modules during robotic-guided walking have been identified. Also, the motor modules controlling lower limb muscles produced variations in muscle activation as a result of the robotic assistance. Module 1 mainly provides body support during the early stance phase. This module increases its contribution in response to increased robotic guidance. Module 2 is a major responsible of leg movement during terminal swing and preparation towards initial stance. Module 3 mainly contributes to control the propulsion of the foot during terminal stance phase. Module 4 provides mainly contribution to control the ankle during initial stance and initial swing. High levels of robot-aided walking (or higher GF) in general induce significantly different muscle activation patterns if compared to treadmill walking, in agreement with results by [16]. These results support the idea that the nervous system may use a modular control strategy and that flexible modulation of module recruitment intensity may be sufficient to meet large changes in mechanical demand.

Our analysis showed that in general there is not a significant difference in the timing provided by the activation signals between robotic-aided walking and treadmill walking when compared at the same walking speed. Nevertheless, we also observed particular conditions with less stereotyped muscle coordination and mechanical output (activation signals and motor modules in robotic-guided walking at 1.5 Km/h speed and with 20% GF), that may not contribute to promote a convenient motor pattern.

In conclusion, the results of this study indicate that the main modular organization of control in physiological walking in healthy humans is in general maintained when adding a GF with a robotic trainer. A low-dimensional, burst-like impulsive control, with activation impulses well timed with respect to the gait phases is in general maintained, with the exception of particular conditions that are uncomfortable for healthy subjects and result in deviations in modules and timing of activations (20% GF and 1.5 Km/h speed). The results indicate that the muscle weightenings can be shaped by changing the GF, according to the view that such weightings during locomotion are more flexible than activation primitives [22]. These results support the idea that robotic guidance does not distort the fundamental control structure in intact physiological pattern and gives strength to the concept that the robotic trainer can be effective in shaping the motor modules with determined conditions of GF and gait speed while maintaining the impulsive control of locomotion. Accordingly, it can be speculated that stroke locomotion rehabilitation with robots may be achieved by shaping motor modules by adjusting GF and speed. This speculation is based on our observation of the control structure during robotic-aided walking and must be confirmed with further research a) including neuro-musculoskeletal models that allow to explain the contribution of muscles and b) to analyse the retention of induced modifications of gait as a function of dose and training intensity [41].

It is still controversial whether if an ischemic event affects motor modules nor their activation signals. Recent research studies led to different results. We distinguish between locomotion and aiming movement: whilst the first one could be mainly exploited at the CPG level, the later should be mainly coded as a combination of supraspinal descending command and a muscle weight coding at spinal level. This scenario is in agreement with the results of Cheung and colleagues [42] (i.e. motor modules may be preserved), since the stroke is a cortical damage that should not interfere with the spinal coding of muscle weightings, once the direction of aiming is given.

The rhythmic activity of locomotion can be imagined as a more decentralized process in which the modulation of muscular activation responds to the integration of peripheral and supraspinal input under the control of the rhythm generating networks of CPG. The three studies (Clark et al. [12], Gizzi et al. [22] and Cheung et al. [42]) agree that a modular organization (of walking and reaching) is shown in stroke patients. Our previous results from Gizzi and colleagues appear different to Clark’s study, but not contradictory: as reported in [12] a central role in the reaction to CVA could be the distance in time from stroke. Whilst in [22] subacute patients were examined, in Clark’s study patients were recruited in their chronic phase. In that work the authors stated that a superimposition of motor modules from healthy controls can happen as an adaptation to stroke. This result was not reported for subacute patients, but both studies agree that the activation signals, although for chronic patient may be also collapsed, may be maintained. Under these premises, it is reasonable to consider that there is an adaptation of stroke patients to cope with a (partly) disrupted contribution from supraspinal centers in the restoration of healthy-like motor modules.

## Conclusion

In conclusion, if motor modules are modified in stroke with maintenance of the activation impulses, robot therapy can be more adequately controlled. The results of this study provide the basis for proposing a novel closed-loop control strategy for intensive gait training in which robotic trainer parameters (GF and gait speed) could be optimally controlled directly exploring the motor protocol of the patient to shape the modular control of synergistic muscles, inducing the required timing of activity generated by central pattern generators. Further work with personalized neuro-musculoskeletal models is required to verify the contribution of investigated muscles to net torque taking into account the learning effect on the training time [43]. Also, such models are to be applied to compute the interaction torques from the commonly available kinetic information in therapeutic exoskeletons. It should be kept in mind that gait is the result of very complex interactions. Any planning efforts to design robot therapy to develop motor modules will help to determine whether the capacity of a central pattern generator characteristic may come to surface when appropriate sensory experience is provided or might be a developmentally determined function of restricted neuronal circuits [44].

## Abbreviations

GF: Guidance force; EMG: Electromyography; fMRI: Functional magnetic resonance imaging; CNS: Central nervous system; NNMF: Nonnegative matrix factorization; BWS: Body weight support; RF: Rectus femoris; VL: Vastus lateralis; ST: Semitendinosus; BF: Biceps femoris; GM: Gastrocnemius medialis; GL: Gastrocnemius lateralis; TA: Tibialis anterior; SENIAM: Surface electromyography for the non-invasive assessment of muscles; DC: Direct current; RMS: Root mean squared; EMG0: initial EMG; EMGr: reconstructed EMG; VAF: Variability accounted for; ROM: Range of motion; ROF: Range of forces; CVA: Cerebrovascular accident.

## Competing interests

The authors declare that they have no competing interests.

## Authors’ contributions

JCM, DF participated in the study concept and design. FB, JCM carried out the setting of procedures and experimentations. FB, JCM performed the statistical analysis. FB, LG, MM, JCM participated in the *analysis and interpretation of published work*. JCM, FB coordinated the drafting of the manuscript. DF, LG, CS, JLP, MM participated in the critical revision for important intellectual content. JCM coordinated and supervised the study. All authors read and approved the final version of the manuscript.

## Supplementary Material

Additional file 1 **Appendix of Tables of results of statistical analysis – Tables present values of correlation of activation signals among all the conditions of GF and speed in the average group and values of correlation of motor modules among all the conditions of GF and speed in the average group.** (file available at http://www.car.upm-csic.es/bioingenieria/better/A1104/Appendix1.pdf). Click here for file
