# Supplementary material for: Effects of robotic guidance on the coordination of locomotion
Source: J Neuroeng Rehabil. 2013 Jul 19;10:79. doi: 10.1186/1743-0003-10-79 (PMC3724716; doi:10.1186/1743-0003-10-79)
Supplement: Additional file 1 — Appendix of Tables of results of statistical analysis – Tables present values of correlation of activation signals among all the conditions of GF and speed in the average group and values of correlation of motor modules among all the conditions of GF and speed in the average group. (file available at http://www.car.upm-csic.es/bioingenieria/better/A1104/Appendix1.pdf). [file 1743-0003-10-79-S1.pdf]

Table 1 - Evaluation of Module 1. ‘Tr.’ represents treadmill walking and ‘Lo.’ represents robotic-guided walking. (A) Correlation of Activation signal 1 among all the conditions of guidance force and speed in the average group. (B) Correlation of Module 1 among all the conditions of guidance force and speed in the average group.

A) Activation signal 1

	Tr. 1.5 Km/h speed	Tr. 2.0 Km/h speed	Tr. 2.5 Km/h speed	Lo. 1.5 Km/h speed 20% GF	Lo. 2.0 Km/h speed 20% GF	Lo. 2.5 Km/h speed 20% GF	Lo. 1.5 Km/h speed 40% GF	Lo. 2.0 Km/h speed 40% GF	Lo. 2.5 Km/h speed 40% GF	Lo. 1.5 Km/h speed 70% GF	Lo. 2.0 Km/h speed 70% GF	Lo. 2.5 Km/h speed 70% GF	Lo. 1.5 Km/h speed 100% GF	Lo. 2.0 Km/h speed 100% GF	Lo. 2.5 Km/h speed 100% GF
**Tr. 1.5 Km/h speed**	1														
**Tr. 2.0 Km/h speed**	,494	1													
**Tr. 2.5 Km/h speed**	,733	,850*	1												
**Lo. 1.5 Km/h speed 20% GF**	,550	-,034	,030	1											
**Lo. 2.0 Km/h speed 20% GF**	,636	,886**	,730	,364	1										
**Lo. 2.5 Km/h speed 20% GF**	,571	,872*	,685	,161	,949**	1									
**Lo. 1.5 Km/h speed 40% GF**	,762*	,764*	,656	,571	,957**	,879**	1								
**Lo. 2.0 Km/h speed 40% GF**	,857*	,834*	,849*	,361	,922**	,892**	,934**	1							
**Lo. 2.5 Km/h speed 40% GF**	,818*	,861*	,845*	,247	,900**	,906**	,901**	,988**	1						
**Lo. 1.5 Km/h speed 70% GF**	,752	,800*	,756*	,447	,959**	,895**	,956**	,958**	,921**	1					
**Lo. 2.0 Km/h speed 70% GF**	,758*	,869*	,848*	,247	,928**	,913**	,900**	,979**	,974**	,967**	1				
**Lo. 2.5 Km/h speed 70% GF**	,727	,920**	,846*	,197	,944**	,942**	,905**	,974**	,985**	,942**	,987**	1			
**Lo. 1.5 Km/h speed 100% GF**	,723	,897**	,779*	,290	,971**	,965**	,948**	,967**	,971**	,941**	,958**	,984**	1		
**Lo. 2.0 Km/h speed 100% GF**	,682	,835*	,812*	,278	,897**	,828*	,866*	,920**	,902**	,957**	,968**	,935**	,887**	1	
**Lo. 2.5 Km/h speed 100% GF**	,797*	,869*	,807*	,264	,899**	,902**	,908**	,965**	,985**	,882**	,932**	,966**	,974**	,842*	1

* Correlation is significant at the 0.05 level (2-tailed).
** Correlation is significant at the 0.01 level (2-tailed).

B) Module 1

**Tr. 1.5 Km/h speed**	1

	Tr. 1.5 Km/h speed	Tr. 2.0 Km/h speed	Tr. 2.5 Km/h speed	Lo. 1.5 Km/h speed 20% GF	Lo. 2.0 Km/h speed 20% GF	Lo. 2.5 Km/h speed 20% GF	Lo. 1.5 Km/h speed 40% GF	Lo. 2.0 Km/h speed 40% GF	Lo. 2.5 Km/h speed 40% GF	Lo. 1.5 Km/h speed 70% GF	Lo. 2.0 Km/h speed 70% GF	Lo. 2.5 Km/h speed 70% GF	Lo. 1.5 Km/h speed 100% GF	Lo. 2.0 Km/h speed 100% GF	Lo. 2.5 Km/h speed 100% GF
**Tr. 2.0 Km/h speed**	,889**	1													
**Tr. 2.5 Km/h speed**	,759*	,902**	1												
**Lo. 1.5 Km/h speed 20% GF**	,899**	,827*	,873*	1											
**Lo. 2.0 Km/h speed 20% GF**	,993**	,895**	,738	,865*	1										
**Lo. 2.5 Km/h speed 20% GF**	,980**	,916**	,765*	,854*	,995**	1									
**Lo. 1.5 Km/h speed 40% GF**	,974**	,872*	,699	,818*	,991**	,991**	1								
**Lo. 2.0 Km/h speed 40% GF**	,909**	,818*	,820*	,895**	,883**	,871*	,878**	1							
**Lo. 2.5 Km/h speed 40% GF**	,972**	,876**	,838*	,953**	,950**	,937**	,928**	,972**	1						
**Lo. 1.5 Km/h speed 70% GF**	,957**	,898**	,873*	,969**	,946**	,945**	,925**	,941**	,986**	1					
**Lo. 2.0 Km/h speed 70% GF**	,967**	,895**	,783*	,896**	,979**	,984**	,978**	,893**	,951**	,972**	1				
**Lo. 2.5 Km/h speed 70% GF**	,973**	,895**	,852*	,955**	,961**	,956**	,943**	,958**	,995**	,996**	,973**	1			
**Lo. 1.5 Km/h speed 100% GF**	,992**	,847*	,710	,875**	,977**	,953**	,955**	,909**	,963**	,929**	,933**	,953**	1		
**Lo. 2.0 Km/h speed 100% GF**	,854*	,599	,578	,896**	,808*	,758*	,760*	,798*	,872*	,846*	,795*	,852*	,871*	1	
**Lo. 2.5 Km/h speed 100% GF**	,972**	,915**	,875**	,948**	,959**	,957**	,938**	,956**	,992**	,990**	,963**	,996**	,953**	,825*	1

* Correlation is significant at the 0.05 level (2-tailed).
** Correlation is significant at the 0.01 level (2-tailed).

Table 2 - Evaluation of Module 2. ‘Tr.’ represents treadmill walking and ‘Lo.’ represents robotic-guided walking. (A) Correlation of Activation signal 2 among all the conditions of guidance force and speed in the average group. (B) Correlation of Module 2 among all the conditions of guidance force and speed in the average group.

A) Activation signal 2

**Tr. 1.5 Km/h speed**	1	
**Tr. 2.0 Km/h speed**	,851*	1

	Tr. 1.5 Km/h speed	Tr. 2.0 Km/h speed	Tr. 2.5 Km/h speed	Lo. 1.5 Km/h speed 20% GF	Lo. 2.0 Km/h speed 20% GF	Lo. 2.5 Km/h speed 20% GF	Lo. 1.5 Km/h speed 40% GF	Lo. 2.0 Km/h speed 40% GF	Lo. 2.5 Km/h speed 40% GF	Lo. 1.5 Km/h speed 70% GF	Lo. 2.0 Km/h speed 70% GF	Lo. 2.5 Km/h speed 70% GF	Lo. 1.5 Km/h speed 100% GF	Lo. 2.0 Km/h speed 100% GF	Lo. 2.5 Km/h speed 100% GF
**Tr. 2.5 Km/h speed**	,804*	,994**	1												
**Lo. 1.5 Km/h speed 20% GF**	,905**	,622	,575	1											
**Lo. 2.0 Km/h speed 20% GF**	,584	,888**	,922**	,339	1										
**Lo. 2.5 Km/h speed 20% GF**	,416	,786*	,844*	,187	,924**	1									
**Lo. 1.5 Km/h speed 40% GF**	,901**	,895**	,876**	,783*	,804*	0,586	1								
**Lo. 2.0 Km/h speed 40% GF**	,536	,883**	,912**	,255	,976**	,920**	,73	1							
**Lo. 2.5 Km/h speed 40% GF**	,047	,492	,553	-,233	,659	,851*	,146	,744	1						
**Lo. 1.5 Km/h speed 70% GF**	,41	,808*	,863*	,157	,960**	,974**	,633	,955**	,792*	1					
**Lo. 2.0 Km/h speed 70% GF**	,357	,757*	,814*	,076	,899**	,987**	,515	,913**	,901**	,966**	1				
**Lo. 2.5 Km/h speed 70% GF**	,128	,567	,637	-,136	,743	,925**	,254	,789*	,979**	,872*	,957**	1			
**Lo. 1.5 Km/h speed 100% GF**	,571	,821*	,847*	,301	,786*	,893**	,549	,818*	,828*	,818*	,907**	,863*	1		
**Lo. 2.0 Km/h speed 100% GF**	,318	,684	,734	,06	,733	,914**	,345	,788*	,938**	,843*	,938**	,961**	,950**	1	
**Lo. 2.5 Km/h speed 100% GF**	,209	,579	,63	-,079	,65	,855*	,22	,715	,958**	,775*	,906**	,961**	,921**	,981**	1

* Correlation is significant at the 0.05 level (2-tailed).
** Correlation is significant at the 0.01 level (2-tailed).

B) Module 2

**Tr. 1.5 Km/h speed**	1					
**Tr. 2.0 Km/h speed**	,902**	1				
**Tr. 2.5 Km/h speed**	,907**	,997**	1			
**Lo. 1.5 Km/h speed 20% GF**	,917**	,710	,739	1		
**Lo. 2.0 Km/h speed 20% GF**	,713	,919**	,903**	,474	1	
**Lo. 2.5 Km/h speed 20% GF**	,712	,918**	,896**	,449	,994**	1

	Tr. 1.5 Km/h speed	Tr. 2.0 Km/h speed	Tr. 2.5 Km/h speed	Lo. 1.5 Km/h speed 20% GF	Lo. 2.0 Km/h speed 20% GF	Lo. 2.5 Km/h speed 20% GF	Lo. 1.5 Km/h speed 40% GF	Lo. 2.0 Km/h speed 40% GF	Lo. 2.5 Km/h speed 40% GF	Lo. 1.5 Km/h speed 70% GF	Lo. 2.0 Km/h speed 70% GF	Lo. 2.5 Km/h speed 70% GF	Lo. 1.5 Km/h speed 100% GF	Lo. 2.0 Km/h speed 100% GF	Lo. 2.5 Km/h speed 100% GF
**Lo. 1.5 Km/h speed 40% GF**	,931**	,983**	,986**	,791*	,875*	,860*	1								
**Lo. 2.0 Km/h speed 40% GF**	,591	,863*	,842*	,328	,980**	,976**	,809*	1							
**Lo. 2.5 Km/h speed 40% GF**	,643	,860*	,832*	,373	,983**	,990**	,799*	,974**	1						
**Lo. 1.5 Km/h speed 70% GF**	,727	,928**	,900**	,444	,948**	,972**	,868*	,945**	,950**	1					
**Lo. 2.0 Km/h speed 70% GF**	,764*	,954**	,935**	,495	,952**	,971**	,892**	,935**	,936**	,991**	1				
**Lo. 2.5 Km/h speed 70% GF**	,733	,939**	,917**	,462	,987**	,995**	,885**	,973**	,976**	,985**	,985**	1			
**Lo. 1.5 Km/h speed 100% GF**	,752	,942**	,920**	,484	,974**	,991**	,881**	,953**	,969**	,990**	,993**	,995**	1		
**Lo. 2.0 Km/h speed 100% GF**	,716	,912**	,889**	,458	,981**	,995**	,849*	,961**	,986**	,976**	,975**	,988**	,993**	1	
**Lo. 2.5 Km/h speed 100% GF**	,770*	,947**	,924**	,500	,980**	,992**	,893**	,953**	,972**	,984**	,986**	,996**	,997**	,988**	1

* Correlation is significant at the 0.05 level (2-tailed).
** Correlation is significant at the 0.01 level (2-tailed).

Table 3 – Evaluation of Module 3. 'Tr.' represents treadmill walking and 'Lo.' represents robotic-guided walking. (A) Correlation of Activation signal 3 among all the conditions of guidance force and speed in the average group. (B) Correlation of Module 3 among all the conditions of guidance force and speed in the average group..

A) Activation signal 3

**Tr. 1.5 Km/h speed**	1							
**Tr. 2.0 Km/h speed**	,997**	1						
**Tr. 2.5 Km/h speed**	,993**	,997**	1					
**Lo. 1.5 Km/h speed 20% GF**	,963**	,953**	,960**	1				
**Lo. 2.0 Km/h speed 20% GF**	,977**	,977**	,961**	,928**	1			
**Lo. 2.5 Km/h speed 20% GF**	,943**	,946**	,921**	,861*	,984**	1		
**Lo. 1.5 Km/h speed 40% GF**	,945**	,944**	,921**	,847*	,976**	,979**	1	
**Lo. 2.0 Km/h speed**	,834*	,851*	,821*	,681	,896**	,932**	,951**	1

	Tr. 1.5 Km/h speed	Tr. 2.0 Km/h speed	Tr. 2.5 Km/h speed	.o. 1.5 Km/h speed 20% GF	.o. 2.0 Km/h speed 20% GF	Lo. 2.5 Km/h speed 20% GF	Lo. 1.5 Km/h speed 40% GF	Lo. 2.0 Km/h speed 40% GF	.o. 2.5 Km/h speed 40% GF	.o. 1.5 Km/h speed 70% GF	.o. 2.0 Km/h speed 70% GF	Lo. 2.5 Km/h speed 70% GF	o. 1.5 Km/h speed 100% GF	o. 2.0 Km/h speed 100% GF	Lo. 2.5 Km/h speed 100% GF
**40% GF**															
**Lo. 2.5 Km/h speed 40% GF**	,894**	,908**	,886**	,784*	,950**	,978**	,964**	,972**	1						
**Lo. 1.5 Km/h speed 70% GF**	,935**	,940**	,919**	,825*	,968**	,978**	,996**	,971**	,979**	1					
**Lo. 2.0 Km/h speed 70% GF**	,849*	,860*	,831*	,697	,904**	,940**	,966**	,995**	,971**	,980**	1				
**Lo. 2.5 Km/h speed 70% GF**	,888**	,909**	,892**	,759*	,921**	,933**	,951**	,981**	,972**	,974**	,973**	1			
**Lo. 1.5 Km/h speed 100% GF**	,935**	,942**	,917**	,821*	,968**	,985**	,990**	,970**	,979**	,995**	,975**	,971**	1		
**Lo. 2.0 Km/h speed 100% GF**	,940**	,950**	,928**	,860*	,976**	,990**	,956**	,927**	,975**	,963**	,923**	,943**	,978**	1	
**Lo. 2.5 Km/h speed 100% GF**	,890**	,913**	,901**	,797*	,935**	,948**	,928**	,946**	,985**	,952**	,934**	,974**	,951**	,964**	1

* Correlation is significant at the 0.05 level (2-tailed).
** Correlation is significant at the 0.01 level (2-tailed).

B) Module 3

**Tr. 1.5 Km/h speed**	1										
**Tr. 2.0 Km/h speed**	,970**	1									
**Tr. 2.5 Km/h speed**	,931**	,984**	1								
**Lo. 1.5 Km/h speed 20% GF**	,929**	,980**	,972**	1							
**Lo. 2.0 Km/h speed 20% GF**	,999**	,977**	,939**	,935**	1						
**Lo. 2.5 Km/h speed 20% GF**	,990**	,952**	,906**	,906**	,988**	1					
**Lo. 1.5 Km/h speed 40% GF**	,996**	,962**	,911**	,913**	,997**	,984**	1				
**Lo. 2.0 Km/h speed 40% GF**	,965**	,993**	,982**	,974**	,973**	,938**	,957**	1			
**Lo. 2.5 Km/h speed 40% GF**	,963**	,984**	,979**	,973**	,968**	,930**	,951**	,996**	1		
**Lo. 1.5 Km/h speed 70% GF**	,960**	,991**	,988**	,988**	,965**	,939**	,943**	,993**	,993**	1	
**Lo. 2.0 Km/h speed 70% GF**	,970**	,986**	,976**	,983**	,973**	,947**	,955**	,992**	,995**	,997**	1

	Tr. 1.5 Km/h speed	Tr. 2.0 Km/h speed	Tr. 2.5 Km/h speed	Lo. 1.5 Km/h speed 20% GF	Lo. 2.0 Km/h speed 20% GF	Lo. 2.5 Km/h speed 20% GF	Lo. 1.5 Km/h speed 40% GF	Lo. 2.0 Km/h speed 40% GF	Lo. 2.5 Km/h speed 40% GF	Lo. 1.5 Km/h speed 70% GF	Lo. 2.0 Km/h speed 70% GF	Lo. 2.5 Km/h speed 70% GF	Lo. 1.5 Km/h speed 100% GF	Lo. 2.0 Km/h speed 100% GF	Lo. 2.5 Km/h speed 100% GF
**Lo. 2.5 Km/h speed 70% GF**	,965**	,990**	,982**	,983**	,971**	,939**	,952**	,997**	,998**	,998**	,998**	1			
**Lo. 1.5 Km/h speed 100% GF**	,984**	,990**	,970**	,972**	,988**	,963**	,975**	,994**	,994**	,992**	,997**	,996**	1		
**Lo. 2.0 Km/h speed 100% GF**	,980**	,935**	,912**	,873*	,974**	,966**	,971**	,928**	,931**	,923**	,934**	,926**	,951**	1	
**Lo. 2.5 Km/h speed 100% GF**	,960**	,989**	,988**	,981**	,965**	,931**	,944**	,996**	,998**	,998**	,997**	,999**	,993**	,928**	1

* Correlation is significant at the 0.05 level (2-tailed).
** Correlation is significant at the 0.01 level (2-tailed).

Table 4 - Evaluation of Module 4. ‘Tr.’ represents treadmill walking and ‘Lo.’ represents robotic-guided walking. (A) Correlation of Activation signal 4 among all the conditions of guidance force and speed in the average group. (B) Correlation of Module 4 among all the conditions of guidance force and speed in the average group..

A) Activation signal 4

**Tr. 1.5 Km/h speed**	1												
**Tr. 2.0 Km/h speed**	,941**	1											
**Tr. 2.5 Km/h speed**	,846*	,881**	1										
**Lo. 1.5 Km/h speed 20% GF**	,413	,557	,310	1									
**Lo. 2.0 Km/h speed 20% GF**	,741	,914**	,869*	,555	1								
**Lo. 2.5 Km/h speed 20% GF**	,306	,418	,734	-,125	,633	1							
**Lo. 1.5 Km/h speed 40% GF**	,784*	,797*	,631	,526	,714	,289	1						
**Lo. 2.0 Km/h speed 40% GF**	,233	,133	,543	-,509	,186	,792*	,112	1					
**Lo. 2.5 Km/h speed 40% GF**	,285	,331	,725	-,182	,493	,948**	,132	,867*	1				
**Lo. 1.5 Km/h speed 70% GF**	,061	-,049	,401	-,569	,021	,715	-,063	,977**	,830*	1			
**Lo. 2.0 Km/h speed 70% GF**	,286	,263	,668	-,351	,374	,912**	,157	,964**	,966**	,924**	1		
**Lo. 2.5 Km/h speed 70% GF**	,359	,385	,755*	-,206	,519	,957**	,238	,897**	,987**	,836*	,981**	1	
**Lo. 1.5 Km/h speed**	,068	-,006	,450	-,515	,095	,784*	-,055	,971**	,892**	,985**	,957**	,895**	1

	Tr. 1.5 Km/h speed	Tr. 2.0 Km/h speed	Tr. 2.5 Km/h speed	). 1.5 Km/h speed 20% (	.o. 2.0 Km/h speed 20% GF	.o. 2.5 Km/h speed 20% GF	.o. 1.5 Km/h speed 40% GF	.o. 2.0 Km/h speed 40% GF	). 2.5 Km/h speed 40% (	Lo. 1.5 Km/h speed 70% GF	.o. 2.0 Km/h speed 70% GF	o. 2.5 Km/h speed 70% GF	o. 1.5 Km/h speed 100% GF	Lo. 2.0 Km/h speed 100% GF	Lo. 2.5 Km/h speed 100% GF
100% GF															
**Lo. 2.0 Km/h speed 100% GF**	,108	,088	,533	-,432	,206	,788*	-,200	,869*	,921**	,887**	,913**	,882**	,919**	1	
**Lo. 2.5 Km/h speed 100% GF**	,397	,362	,750	-,240	,429	,879**	,192	,927**	,968**	,880**	,981**	,978**	,923**	,912**	1

* Correlation is significant at the 0.05 level (2-tailed).
** Correlation is significant at the 0.01 level (2-tailed).

B) Module 4

**Tr. 1.5 Km/h speed**	1														
**Tr. 2.0 Km/h speed**	,941**	1													
**Tr. 2.5 Km/h speed**	,620	,726	1												
**Lo. 1.5 Km/h speed 20% GF**	,831*	,635	,463	1											
**Lo. 2.0 Km/h speed 20% GF**	,673	,723	,623	,616	1										
**Lo. 2.5 Km/h speed 20% GF**	,683	,771*	,889**	,537	,874*	1									
**Lo. 1.5 Km/h speed 40% GF**	,920**	,915**	,430	,649	,719	,623	1								
**Lo. 2.0 Km/h speed 40% GF**	,625	,726	,989**	,491	,624	,873*	,425	1							
**Lo. 2.5 Km/h speed 40% GF**	,622	,724	,969**	,437	,673	,945**	,483	,948**	1						
**Lo. 1.5 Km/h speed 70% GF**	,705	,754	,949**	,529	,609	,891**	,530	,907**	,965**	1					
**Lo. 2.0 Km/h speed 70% GF**	,778*	,786*	,873*	,623	,702	,918**	,648	,831*	,932**	,970**	1				
**Lo. 2.5 Km/h speed 70% GF**	,661	,714	,962**	,519	,626	,912**	,478	,930**	,983**	,992**	,962**	1			
**Lo. 1.5 Km/h speed 100% GF**	,882**	,934**	,849*	,692	,792*	,852*	,784*	,832*	,812*	,853*	,852*	,823*	1		
**Lo. 2.0 Km/h speed 100% GF**	,635	,757*	,769*	,220	,345	,661	,562	,705	,790*	,854*	,800*	,803*	,733	1	
**Lo. 2.5 Km/h speed 100% GF**	,754	,828*	,979**	,580	,703	,921**	,589	,961**	,964**	,972**	,933**	,969**	,926**	,805*	1

Tr. 1.5 Km/h speed	Tr. 2.0 Km/h speed	Tr. 2.5 Km/h speed	o. 1.5 Km/h speed 20% GF	. 2.0 Km/h speed 20%	o. 2.5 Km/h speed 20% GF	Lo. 1.5 Km/h speed 40% GF	Lo. 2.0 Km/h speed 40% GF	. 2.5 Km/h speed 40%	o. 1.5 Km/h speed 70% GF	o. 2.0 Km/h speed 70% GF	. 2.5 Km/h speed 70%	o. 1.5 Km/h speed 100% GF	o. 2.0 Km/h speed 100% GF	Lo. 2.5 Km/h speed 100% GF

* Correlation is significant at the 0.05 level (2-tailed).
** Correlation is significant at the 0.01 level (2-tailed).
